# The Risk of Iatrogenic Hypercalcemia in Patients Undergoing Calcium Sulphate Beads Implantation in Prosthetic Joint Surgery: A Systematic Review

**DOI:** 10.7759/cureus.18777

**Published:** 2021-10-14

**Authors:** Muhammad Yasir Tarar, Ko Ko Zayar Toe, Komal Javed, Numan Shah, Aizaz Khalid

**Affiliations:** 1 Trauma and Orthopaedics, Salford Royal NHS Foundation Trust, Manchester, GBR; 2 Orthopaedics and Trauma, Salford Royal NHS Foundation Trust, Manchester, GBR; 3 Internal Medicine, Services Institute of Medical Sciences, Lahore, PAK

**Keywords:** iatrogenic hypercalcemia, calcium sulphate beads, total knee replacement (tkr), total hip replacement (thr), peri-prosthetic joint infection

## Abstract

Calcium sulphate beads are increasingly being used in the management of prosthetic joint infections (PJI). Traditionally their use was limited to a void or dead space-filling combined with other additives such as Hydroxyapatite. Over the last decade, they have been developed to act more frequently as an antibiotics delivery system. Stimulan, a bio-absorbable form of Calcium sulfate, theoretically has an increased risk of hypercalcemia. Over the last few years, there have been published case reports which report it as an isolated cause of iatrogenic hypercalcemia. The sparsity of literature on this topic makes it difficult for surgeons to decide on the use of Calcium sulphate beads in patients with hypercalcemia predisposition in conditions like autoimmune disorders, sarcoidosis, malignancy, granulomatous diseases, heterotopic ossification, and hyperparathyroidism. The study was performed to assess the risk of hypercalcemia in patients after Calcium sulphate beads implantation in PJI. Two reviewers searched relevant literature in 3 online databases using cochrane methodology for systematic reviews. Studies reporting complications with the use of calcium sulphate beads in prosthetic joints were included. Studies reporting on less than five patients and studies reporting use in any other surgeries were excluded. The search of databases resulted in a total of 96 articles. After screening, a total of four articles were deemed suitable to be included in the analysis. A total of 1049 patients underwent calcium sulfate beads implantation, out of which 44 (4.2%) reported hypercalcemia with 41 (3.91%) transient in nature and 3 (0.28%) required management, including one with ICU admission. The result of this systematic review shows that calcium sulphate beads are safe and effective against PJI. There is a significant risk of transient hypercalcemia in susceptible patients and a low risk of symptomatic hypercalcemia.

## Introduction and background

The use of calcium sulfate as a bone void filler is over a century old. However, its use remained limited until 1959 when Peltier et al. found that this substance (a component of plaster of Paris) acts as a potent agent for bone filling, allowing bone formation without any foreign body reaction [1]. Traditionally PMMA (Polymethyl methacrylate) was used as an antibiotics carrier system in infections, but the need for removal after surgery has remained a significant downside [2]. The calcium sulfate beads are increasingly used these days as a bone filler and antibiotic delivery system in infections and fractures [3,4]. Several drug combinations, including antifungals, have been used with Calcium sulphate beads for prosthetic joint infections, including vancomycin, gentamycin, tobramycin, amikacin, and caspofungin [5-8]. Over the last decade, several case reports and case series have highlighted the incidence of hypercalcemia with Calcium sulphate beads use [5-9]. There is a lack of evidence of its safety profile in patients with a risk of high serum calcium such as hyperparathyroidism, malignancy, autoimmune disorders, and granulomatous diseases. Hypercalcemia can lead to cardiac arrhythmia, confusion, renal stones, and increased hospital stays [10]. 

This study aims to assess the risk of hypercalcemia using Calcium sulphate beads in prosthetic joint infections. It proposes the consideration of additional monitoring in high-risk patients. Furthermore, if Calcium sulphate beads increase the serum calcium levels, are there any specific needs to monitor serum calcium levels in high-risk patient groups considering serum calcium are not routinely monitored postoperatively.

## Review

Methodology

**Figure 1:**
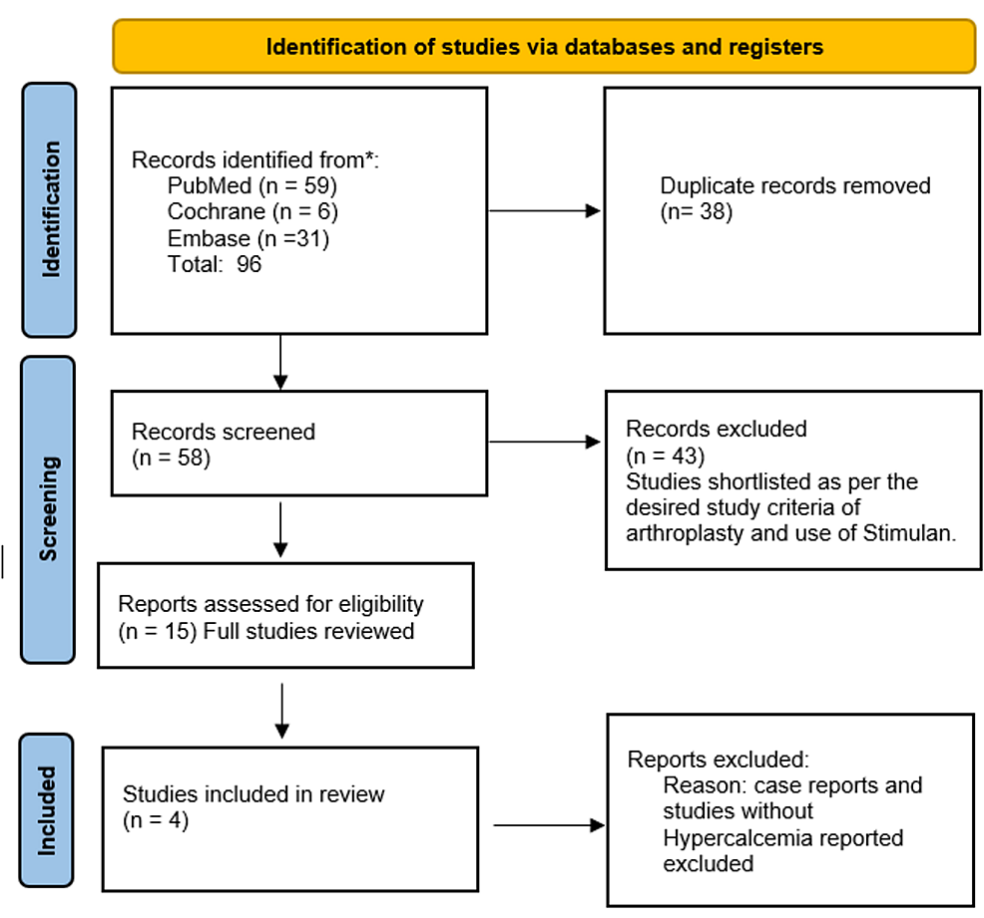
PRISMA study methodology used for the systematic review. [11]

Two reviewers searched relevant literature in 3 online databases using cochrane methodology for systematic reviews. The words "Calcium sulphate beads", "calcium sulfate beads", and "Stimulan" were cross searched with the words "Arthroplasty", "joints", "complication", and "hypercalcemia" in Pubmed, Cochrane, and Embase. A total of 96 articles were found; after the removal of duplications, 58 articles were attained. Two reviewers reviewed the shortlisted articles' titles and abstracts against inclusion criteria. Fifteen full articles were reviewed, and four were selected for the study. Eligibility criteria were any study that reported the use of Calcium sulfate beads in prosthetic joints with mentioning of complications. Disagreements for inclusion were discussed between the reviewers and, if not resolved, discussed with one of the senior authors. The Preferred Reporting Items for Systematic Review and Meta-Analyses (PRISMA) methodology was used [Figure 1] [11]. The design methodology of each study was determined using the guidelines described by Mathes and Pieper [12].

Discussion

Prosthetic joint infection (PJI) is a severe complication with an incidence of 1-2% of primary arthroplasties and is associated with a high morbidity rate [13]. The Musculoskeletal Infection Society (MSIS) proposed diagnostic criteria for Periprosthetic Joint infection, which includes the presence of sinus tract communicating with the prosthesis, isolating a bug, and six laboratory parameters as listed in [Table 1] [14]. The European Bone and Joint Infection Society's (EBJIS) definition of Periprosthetic infection is becoming more popular and is considered more sensitive by various authors [15-17]. Periprosthetic infection is challenging to manage; it impacts patients' lives and has significant economic implications on the healthcare system. A study published in 2020 has shown that following a primary Total Hip Replacement, patients who develop PJI and undergo revision surgery cost over five times (33,000 pounds) more than the patients not undergone revision surgery in inpatient and daycare admissions [18].

**Table 1:** Musculoskeletal Infection Society (MSIS) definition of peri-prosthetic joint infection [14]. ESR: Erythrocyte Sedimentation rate, CRP: C-reactive protein PJI may be present in fewer than four of these criteria are met.

Musculoskeletal Infection Society (MSIS) definition of peri-prosthetic joint infection.
1. There is a sinus tract communicating with the prosthesis; or
2. A pathogen is isolated by culture from at least two separate tissue or fluid samples obtained from the affected prosthetic joint; or
3. Four of the following six criteria exist:
1. Elevated serum erythrocyte sedimentation rate (ESR) and serum C-reactive protein (CRP) concentration,
2. Elevated synovial leukocyte count,
3. Elevated synovial neutrophil percentage (PMN%),
4. Presence of purulence in the affected joint,
5. Isolation of a microorganism in one culture of periprosthetic tissue or fluid, or
6. Greater than five neutrophils per high-power field in five high-power fields observed from histologic analysis of periprosthetic tissue at ×400 magnification.

Staging of the patient has great influence over the possible risk of PJI [Table 2, 3] [19]. Pre-operative optimization of modifiable risks of those factors is associated with improved overall outcomes. There are several pre-operative bundle protocols; however, several variables are consistently present across the varying protocols: such as BMI (<35 kg/m2), Haemoglobin (>11 - 12 g/dl), Glucose control (e.g. HbA1C < 7.0 - 7.5 %), no tobacco use for > 30 days, MRSA (Methicillin-resistant Staphylococcus aureus colonization status) and nutritional status as indicated by albumin (>3.0 - 3.5 g/dl) [20].

**Table 2:** A staging system for prosthetic joint infection risk (part 1) [19] ﻿Stage = infection type + systemic host grade + local extremity grade; e.g I-A-1, III-B-2

Category	Grading	Description
Infection Type	I	Early postoperative infection (< 4 weeks postoperatively)
	II	Hematogenous infection (< 4 weeks' duration)
	III	Late chronic infection (> 4 weeks' duration)
Systemic host grade	A	Uncompromised (no compromising factors)
	B	Compromised (1 to 2 compromising factors)
	C	Significant compromise (> 2 compromising factors) OR one of the following: - Absolute neutrophil count <1000 - CD4 t cell count < 100 - Intravenous drug abuse - Chronic active infection at other site - Dysplasia/neoplasm of immune system (e.g. Myelodysplasia, CLL)
Local extremity grade	1	Uncompromised (no compromising factors
	2	Compromised (1-2 compromising factors)
	3	Significant compromise (> 2 compromising factors)

**Table 3:** A staging system for prosthetic joint infection risk (part 2) [19]

Systemic Host- Compromising factors host (Medical and immune)
Age ≥ 80 yrs
Alcoholism
Chronic active dermatitis or cellulitis
Chronic indwelling catheter
Chronic malnutrition (albumin < 3.0gm/dl)
Current nicotine use (inhalational or oral)
Diabetes (requiring oral agents and/or insulin)
Hepatic insufficiency (cirrhosis)
Immunosuppressive drugs (e.g., methotrexate, prednisone, cyclosporine)
Malignancy (history of, or active)
Pulmonary insufficiency (room air arterial blood gas O2 < 60%)
Renal failure requiring dialysis
Systemic inflammatory disease (rheumatoid arthritis, systemic lupus erythematosus)
Systemic immune compromise from infection or disease (human immunodeficiency virus, acquired immunodeficiency virus)
Local Extremity Grade (wound) - Compromising factors
Active infection present > 3-4 months
Multiple incisions (creating skin bridges)
Soft tissue loss from prior trauma
Subcutaneous abscess >8 cm2
Synovial cutaneous fistula
Prior periarticular fracture or trauma about joint (especially crush injury)
Prior local irradiation to wound area
Vascular insufficiency to an extremity: (absent extremity pulses, chronic venous stasis disease, calcific arterial disease)

A principle of treatment option for all PJI is thorough debridement and adequate delivery of antibiotics to the affected joint space. Surgical treatment options for PJI include single-stage revision (2 in 1), two-stage revision, and DAIR procedure (Debridement and Implant retention, which involved exchanging the modular components of the affected prosthesis). The latter procedure included the implantation of calcium sulfate beads and intravenous antibiotics [17]. Although the best surgical approach for all cases of PJI cannot be identified, it is universally accepted that antibiotics play an essential role in the management of PJI. Generally, long-term antibiotics are effective; however, in cases of PJI, local devascularization of infected tissues can prevent local antibiotic delivery.

Moreover, in chronic scenarios, biofilm formation protects the pathogen(s) from the bactericidal action of antibiotics [21]. In this case, local delivery systems offer a solution. A study has shown that antibiotic-impregnated Calcium sulphate hemihydrate is a useful tool for local delivery of therapeutic concentrations. Calcium sulphate acts as a mineral phase of bone, and they are absorbed at a rate similar to bone formation; thus, they provide structural support and prevent fibrous tissue ingrowth [23]. They act as a vehicle for local high-dose antibiotic delivery. In each of their five postoperative days, they are evaluated with Tobramycin and Vancomycin, and mean local concentrations exceeded the minimum amount of antibiotic needed to inhibit common pathogens [22].

The use of calcium sulphate inside the body is not a new phenomenon [23]. The first internal use was reported by Dressmann et al. in 1892 when they used gypsum(calcium sulphate dihydrate) to fill body defects in 8 patients [24]. Peltier first documented the concern of hypercalcemia by implantation of absorbable calcium compounds in 1969. He observed an initial rise in calcium levels in dogs in the postoperative period, a finding that was not later demonstrated on patients [1]. It has been hypothesized that hypercalcemia following placement of calcium sulphate compounds is due to a rapid uptake of calcium ions from dilated capillary beds, so-called 'dumping' of calcium into the systemic circulation. This occurrence is poorly understood and is thought to be brought on by a combination of host factors [7].

In our review, we identified 1049 patients undergoing revision arthroplasty. The predominant indication was PJI. Transient hypercalcemia was identified in 44 (4.2%) of these patients collectively. 3 (0.28%) of these cases developed symptomatic hypercalcemia, requiring one ICU admission therapy. Forty-one cases did not need any calcium-specific therapy.

Evidence of iatrogenic hypercalcemia from the use of Stimulan is also found in multiple case reports [25-27]. In one of these cases reported by Jung et al. [9], the serum calcium rose to 5.41mmol/l. While three of these four cases were treated with fluid resuscitation and calcitonin, one patient developed renal failure and anuria [27]. This patient had to undergo multiple sessions of hemodialysis for 22 days, followed by recovery. In 2013, McPherson et al. [5] conducted an extensive analysis of 250 patients undergoing revision arthroplasty. They aimed to review complications arising from using calcium sulphate beads as a therapeutic and preventive modality. They found these devices were associated with a complication rate of 11.6%, including wound drainage (3.2%) and heterogenous ossification (1.2%). However, no cases of hypercalcemia or hypercalcemia-associated symptoms were reported in this study [Table 5].

**Table 4:** Demographics of selected studies with antibiotic combinations used, procedures performed and follow-up period [5-8] THA- Total hip arthroplasty; DAIR- debridement, antibiotics, irrigation, and retention; TKA- Total knee arthroplasty

Article by	Study method	Study type	Age mean	Sex (M: F)	Antibiotic combination used	Procedure	Follow up
Sandiford [8]	Prospective	Case series	67	13:16	Vancomycin (n=28) Gentamicin (n=27) Amikacin (n=1) Caspofungin (n=1)	Single- staged revision (n=7) First- stage revision (n=4) Second-stage revision (n=9) DAIR (n=6) Excision arthroplasty (n=1) Periprosthetic fracture (n=2)	6 weeks
Kallala and Haddad [6]	Prospective	Case series	64.8	8:7	1g of vancomycin and 240mg of gentamicin per 10cc of bead mixture with sterile water.	Single–staged revision of a resurfacing arthroplasty of the hip to a THA (2) revision of an infected primary THA (3) infected revision THA (3) infected proximal femoral replacement (1) revision of an infected primary TKA (3) infected revision TKA (3)	Mean 16 months (12 to 22)
Kallal et al [7]	Prospective	Case series	63	374:381	1g of vancomycin mixed with each 10cc of calcium sulphate and 240mg of liquid tobramycin (40mg/ml) was added. in patients with a fungal infection, 50mg of amphotericin b was also added.	Single staged revision of knee (209) DAIR of the knee (49), First of 2 stages of the knee (108), Second of 2 stage knee (90), Single-stage revision of hip (159) DAIR of the hip ( 19), First of 2 stage revision of hip (68), Second of 2 stage revision of hip (53)	Mean 35 months (0 to 78)
McPherson et al [5]	Prospective	Case series	Not stated	Not stated	1gm of Vancomycin powder and 240mg of liquid Tobramycin mixed with 10cc of Stimulan powder	Aspectic revision TKA (66) DECRA TKA (16) Resection TKA (35) Reimplantation TKA (25) Aseptic revision THA (58) DECRA THA (8) Resection THA (24) Reimplantation THA (18)	Minimum of 3 months for all patients maximum of 12 months

The first study to report hypercalcemia in a cohort was conducted by Kallala et al. in 2015 [6]. They prospectively analyzed 15 patients undergoing revision arthroplasty for PJI. They found transient hypercalcemia in 3 (20%) of their cases, with one of these going on to develop symptomatic hypercalcemia. A calcium level of 3.17 mmol/l was recorded on the second postoperative day and was managed with 4 litres of normal saline and 4mg IV Zolendronate over 24 hours. The calcium levels peaked at 3.54 mmol/l on the fifth postoperative day despite ongoing treatment, after which the patient was moved to intensive care and made a full recovery subsequently [Table 5].

Another large study published by Kallala et al. in 2018 examined complications associated with a calcium sulphate antibiotic delivery system [7]. This study enrolled 755 patients undergoing revision arthroplasty and found hypercalcemia in 41 (5.4%) patients. Two of these patients also developed symptoms and were treated with normal saline and intravenous bisphosphonates, followed by the return of calcium to baseline. The mean calcium postoperatively for the hypercalcemic patients was 2.97 mmol/l which returned to baseline on the fifth postoperative day [Table 5].

The latest addition to the literature regarding iatrogenic hypercalcemia was made by Sandiford et al. in 2020 [8]. They prospectively evaluated 29 patients undergoing revision hip arthroplasty for evidence of hypercalcemia. They measured serum calcium levels serially and found that the mean calcium of their cohort increased from 2.5 mmol/l preoperatively to 2.7 mmol/l on the first postoperative day (p=0.09). This increase was sustained in the first two weeks, after which the mean calcium levels returned to 2.4mmol/l six weeks postoperatively. No patients in this cohort developed symptomatic or transient hypercalcemia, and none needed treatment. Note that the reference range of calcium for this study was <3 mmol/l [Table 5].

**Table 5:** The volume of Calcium sulphate beads used in Joints including Hips and Knees with Hypercalcemia correlation and management [5-8]

Author	Calcium sulphate derivative /dose	Total joints	Knees (n)	Hips (n)	Other joints	Hypercalcaemia	Calcium value	Transient Hypercalcemia	Long-standing Hypercalcemia	Management	Space Calcium sulphate used in
Sandiford [8]	Stimulan Mean volume (25ml) Range of 20-40ml	29	0	29	0	Not specified	Mean preoperative corrected serum calcium level was 2.5mmol/l. Day 1 post surgery was 2.7 mmol/l. For week 1, week 2 and week 6 the mean values were 2.7 mmol/l, 2.7 mmol/l and 2.4 mmol/l respectively.	Not specified	0	No treatment	Intracapsular compartment of the hip
Kallala and Haddad [6]	Stimulan Maximum of 40 cc	15	6	9	0	3	Not stated	3	0	1 patient developed symptoms and required rehydration with four litres of IV 0.9% saline over 24-hours and IV bisphosphonates (4 mg of Zoledronic acid over 15 minutes)	Around the hip and knee joint
Kallal et al [7]	Stimulan mean of 23.39 cc (5 to 80)	755	456	299	0	41	the mean levels were 11.7 mg/dl (10.8 to 14.9) the levels returned to normal at a maximum of five days postoperatively.	41	0	two patients in the revision THA group developed symptoms and were treated with one iv dose of bisphosphonate and 0.9% saline.	For patients undergoing knee surgery, the beads were placed in the medial and lateral gutters. For those under-going hip surgery, they were placed deeply, inferior to the acetabulum and around the proximal femur.
McPherson et al [5]	Stimulan ranged Volume 5cc to 70cc in hip cases and 5cc to 50cc in knee	250	142	108	0	0	n/a	0	0	n/a	For knee cases, the Stimulan beads were delivered along the medial and lateral gutters of the knee For hip cases, the Stimulan antibiotics beads were delivered into the deep hip space inferior to the acetabulum and around the proximal femur.

The risk of hypercalcemia has been proposed to be linked with the amount of calcium sulphate used [7,8]. Our selected studies have used variable concentrations of Calcium sulphate antibiotic carrier Stimulan with a mean volume of 25ml of Stimulan (20-40ml) used by Sandiford [8], 23.39 ml (5-80 ml) in Kallala et al. [7]. The manufacturers of Stimulan recommend the dosage of 20cc to be used. However, in our review, we found that these recommendations were mostly exceeded. The isolated case of ICU admission following symptomatic hypercalcemia was administered 40cc of the product. Kallala used a maximum of 40cc and Haddad et al. [6], whereas McPherson et al. used a range of 5-70cc in hip cases and 5-50cc in knee cases [Table 5] [5]. 

The procedural spaces used for the implantation of calcium sulphate beads can also potentially be related to calcium absorption. This can be related to variable vascular patterns in and around a joint. In hip surgery, Kallala et al. used the space inferior to the acetabulum and proximal femur for implantation [6,7], while Sandiford chose intracapsular implantation [8]. In knee surgery, medial and lateral gutters were used by both McPherson and Kallala et al. [Table 5] [5-7].

In our literature review regarding iatrogenic hypercalcemia due to calcium sulphate beads, we did not find any attributed mortality. However, significant side effects have been reported, including serious morbidities such as renal failure and symptoms requiring ICU admission. These studies and the theoretical risk of absorption of rapid calcium absorption via capillary beds around the implantation site warrant caution and vigilance on the orthopaedic surgeon and management team.

## Conclusions

Hypercalcemia is a potential complication in patients undergoing calcium sulphate bead implantation in prosthetic joint surgery. This complication is rare in occurrence but can result in morbidity for the patient. Further studies are needed to evaluate this relationship.

## References

[1] Peltier LF, Bickel EY, Lillo R, Thein MS (1957). The use of plaster of paris to fill defects in bone. Ann Surg.

[2] Neut D, van Horn JR, van Kooten TG, van der Mei HC, Busscher HJ (2003). Detection of biomaterial-associated infections in orthopaedic joint implants. Clin Orthop Relat Res.

[3] Helgeson MD, Potter BK, Tucker CJ, Frisch HM, Shawen SB (2009). Antibiotic-impregnated calcium sulfate use in combat-related open fractures. Orthopedics.

[4] McKee MD, Li-Bland EA, Wild LM, Schemitsch EH (2010). A prospective, randomized clinical trial comparing an antibiotic-impregnated bioabsorbable bone substitute with standard antibiotic-impregnated cement beads in the treatment of chronic osteomyelitis and infected nonunion. J Orthop Trauma.

[5] McPherson EJ, Dipane MV, Sherif M (2013). Dissolvable antibiotic beads in treatment of periprosthetic joint infection and revision arthroplasty - the use of synthetic pure calcium sulfate (Stimulan®) impregnated with vancomycin & tobramycin. Reconstructive Review.

[6] Kallala R, Haddad FS (2015). Hypercalcaemia following the use of antibiotic-eluting absorbable calcium sulphate beads in revision arthroplasty for infection. Bone Joint J.

[7] Kallala R, Harris WE, Ibrahim M, Dipane M, McPherson E (2018). Use of Stimulan absorbable calcium sulphate beads in revision lower limb arthroplasty: safety profile and complication rates. Bone Joint Res.

[8] Sandiford NA (2020). Complication rates are low with the use of Stimulan calcium sulphate based antibiotic delivery system in the management of patients with hip-related PJI: early results of a consecutive case series. Hip Int.

[9] Jung Y, Moe K, Torres EA, Kalantar-Zadeh K, Hanna RM (2020). Unique case of profound iatrogenic hypercalcemia in a patient with recent orthopedic prosthetic infection. Clin Nephrol Case Stud.

[10] Sadiq NM, Naganathan S, Badireddy M (2021). Hypercalcemia. https://www.ncbi.nlm.nih.gov/books/NBK430714/.

[11] Liberati A, Altman DG, Tetzlaff J (2009). The PRISMA statement for reporting systematic reviews and meta-analyses of studies that evaluate health care interventions: explanation and elaboration. J Clin Epidemiol.

[12] Mathes T, Pieper D (2017). Clarifying the distinction between case series and cohort studies in systematic reviews of comparative studies: potential impact on body of evidence and workload. BMC Med Res Methodol.

[13] Izakovicova P, Borens O, Trampuz A (2019). Periprosthetic joint infection: current concepts and outlook. EFORT Open Rev.

[14] Parvizi J, Zmistowski B, Berbari EF (2011). New definition for periprosthetic joint infection: from the workgroup of the Musculoskeletal Infection Society. Clin Orthop Relat Res.

[15] Renz N, Yermak K, Perka C, Trampuz A (2018). Alpha defensin lateral flow test for diagnosis of periprosthetic joint infection: not a screening but a confirmatory test. J Bone Joint Surg Am.

[16] McNally M, Sousa R, Wouthuyzen-Bakker M (2021). The EBJIS definition of periprosthetic joint infection. Bone Joint J.

[17] Li C, Renz N, Trampuz A (2018). Management of periprosthetic joint infection. Hip Pelvis.

[18] Garfield K, Noble S, Lenguerrand E, Whitehouse MR, Sayers A, Reed MR, Blom AW (2020). What are the inpatient and day case costs following primary total hip replacement of patients treated for prosthetic joint infection: a matched cohort study using linked data from the National Joint Registry and Hospital Episode Statistics. BMC Med.

[19] McPherson EJ (2002). Periprosthetic total hip infection: outcomes using a staging system. Clin Orthop Relat Res.

[20] Johns WL, Layon D, Golladay GJ, Kates SL, Scott M, Patel NK (2020). Preoperative risk factor screening protocols in total joint arthroplasty: a systematic review. J Arthroplasty.

[21] Costerton JW, Stewart PS, Greenberg EP (1999). Bacterial biofilms: a common cause of persistent infections. Science.

[22] Maale GE, Eager JJ, Mohammadi DK, Calderon FA 2nd (2020). Elution profiles of synthetic CaSO4 hemihydrate beads loaded with vancomycin and tobramycin. Eur J Drug Metab Pharmacokinet.

[23] Tay BK, Patel VV, Bradford DS (1999). Calcium sulfate- and calcium phosphate-based bone substitutes. mimicry of the mineral phase of bone. Orthop Clin North Am.

[24] Dreesmann H (1892). Ueber Knochenplombierung bei Hohlenformigen Defekten des Knochens.

[25] Carlson Jr CR, Markulis E, Thompson E, Havill J (2015). A novel case of hypercalcemia following the use of calcium sulfate beads. Nephrol Open J.

[26] Vora A, Ali S (2019). Prolonged hypercalcemia from antibiotic-eluting calcium sulfate beads. AACE Clin Case Rep.

[27] Magdaleno A, McCauley RA (2019). Severe hypercalcemia after joint arthroscopy: calcium sulfate beads to blame. AACE Clin Case Rep.

